# Efficacy of an Online Weight Loss Program for Mexican Adults: Findings From a Randomized Controlled Trial

**DOI:** 10.1002/osp4.70165

**Published:** 2026-06-19

**Authors:** Alma Lorena Ruelas Yanes, Teresita de Jesús Martínez‐Contreras, Maria del Carmen Candia‐Plata, Julián Esparza‐Romero, Ana Lourdes Mata‐Pineda, Arelly Ornelas‐Vargas, Rolando Giovanni Díaz‐Zavala, Michelle M. Haby

**Affiliations:** ^1^ Departamento de Ciencias Químico‐Biológicas, Facultad Interdisciplinaria de Ciencias Biológicas y de Salud Universidad de Sonora Encinas y Rosales s/n Hermosillo México; ^2^ Departamento de Nutrición Facultad de Medicina Mexicali Universidad Autónoma de Baja California Humberto Torres Sanginés s/n, Centro Cívico Mexicali México; ^3^ Departamento de Medicina y Ciencias de la Salud, Facultad Interdisciplinaria de Ciencias Biológicas y de Salud Universidad de Sonora Encinas y Rosales s/n Hermosillo México; ^4^ Unidad de Investigación en Obesidad y Diabetes Centro de Investigación en Alimentación y Desarrollo A.C. Hermosillo México; ^5^ Departamento de Pesquerías y Biología Marina CONAHCyT‐Instituto Politécnico Nacional‐Centro Interdisciplinario de Ciencias Marinas (IPN‐CICIMAR) Av. Instituto Politécnico Nacional s/n La Paz México; ^6^ School of Population and Global Health The University of Melbourne Melbourne Australia

**Keywords:** intensive lifestyle interventions, Mexico, obesity, weight loss

## Abstract

**Background:**

Obesity is a major public health crisis in Mexico. With internet usage rapidly increasing, digital health interventions present a clear opportunity for treating obesity. This study addressed a key gap in research by evaluating a web‐based Intensive Lifestyle Modification intervention adapted from the Diabetes Prevention Program for weight loss (DPP‐web) in this population.

**Methods:**

To evaluate the efficacy of the DPP‐web in Mexican adults with overweight or obesity at 3 and 6 months compared with a wait‐list control group, a randomized controlled trial (*n* = 62) with two groups was conducted. The primary outcome was the change in weight at 3 months, with secondary outcomes including changes in anthropometric parameters, quality of life and depressive symptoms at three and 6 months, and biochemical measures at 3 months.

**Results:**

Participants, predominantly women (81%), with an average age of 31.5 ± 8.6 years and BMI of 33.4 ± 4.85 kg/m^2^, had retention rates of 77% at 3 months and 60% at 6 months. The treatment effect by intention‐to‐treat analysis was −3.65 kg (95% CI −5.41, −1.89, *p* < 0.01) at 3 months and −5.50 kg (95% CI −7.58, −3.41) at 6 months, favoring the DPP‐group.

**Conclusions:**

The DPP‐web was effective for weight loss in the Mexican population at three and 6 months and could help to improve access to an effective obesity treatment in Spanish‐speaking countries, including Mexico.

## Graphical abstract

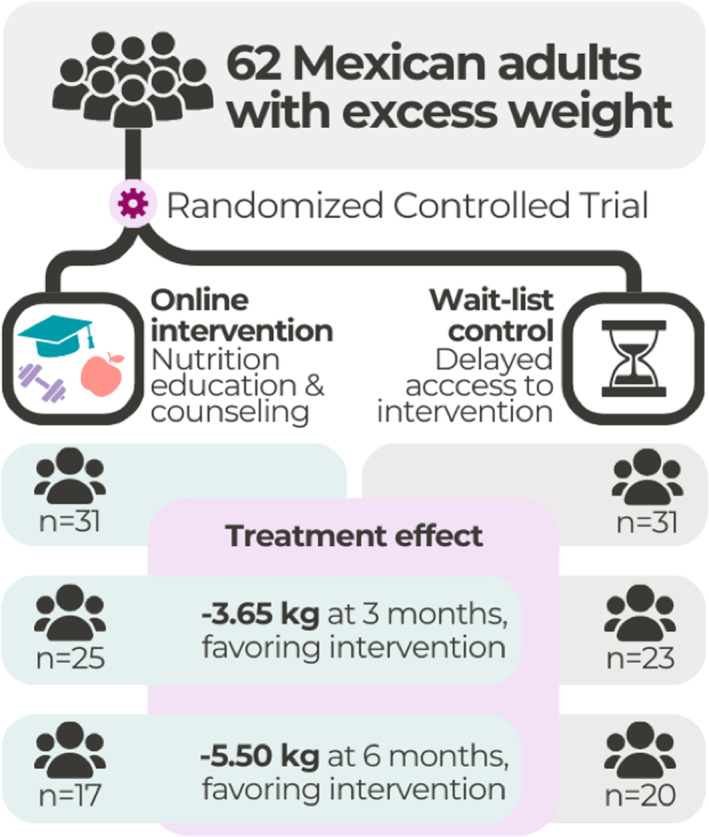

## 1 Introduction

Addressing obesity is a matter of utmost urgency [1], particularly in low and middle‐income countries like Mexico, where 74.5% of the adult population is affected by excess weight [2]. Within this context, Intensive Lifestyle Modification Interventions (ILM) are considered a cornerstone of obesity management, offering a strategy for achieving initial weight loss and improving metabolic health [3]. Although the weight loss achieved may be modest compared to recent pharmacological or surgical advances, ILM provides the necessary behavioral framework that must underpin any evidence‐based treatment [3].

An effective ILM protocol usually involves two to four sessions per month facilitated by trained interventionists that apply cognitive behavioral strategies to improve diet and physical activity [4]. The long‐term impact of ILMs on improving cardiometabolic outcomes has been rigorously assessed [5, 6] with evidence spanning up to 3 decades [7]. Moreover, these interventions have demonstrated effectiveness across various formats, in multiple countries, and within different settings, including digital [8].

One of the most studied ILM protocols is the Diabetes Prevention Program (DPP), which demonstrated that diabetes could be prevented [9]. What sets the DPP apart is its cornerstone “Group Lifestyle Balance” curriculum [10], whose openness to adaptation has played a pivotal role in facilitating the successful translation and evaluation of the DPP in numerous diverse settings, further underscoring its versatility and impact [9, 11, 12].

In 2015, a randomized controlled trial that evaluated a 3‐month in‐person adaptation of the DPP in Mexican adults compared to monthly sessions with a dietitian found a median treatment effect of −5.10 kg (*p* < 0.001) and a 97% retention rate, favoring the intervention group [13]. Similarly, a non‐controlled multicenter study in 5 clinics conducted in Mexico with the same curriculum found a weight loss of between 2.76 (SD 4.76) kg and 7.92 (SD 6.85) kg at 6 months [12]. However, a systematic review found that no trials of online ILM protocols have been conducted in low‐ or middle‐income or Spanish‐speaking countries, such as Mexico [14].

Remote ILM protocols, delivered via the internet, telephone, text messaging, or mobile applications, are effective for weight loss, cost less, and can expand treatment reach compared to in‐person interventions [3]. This modality could benefit those with a lack of access to transport, those who live far away from treatment centers, or those who have work commitments that restrict their ability to attend in‐person appointments during normal work hours. Although their results do not reach the effectiveness of in‐person interventions [15, 16, 17], the quality of the trials has been criticized due to their high risk of bias [15].

Since 86.5% of Mexicans have access to the internet [18] and BMI is rapidly increasing in Latin America [19], digitally delivered ILM protocols could help to mitigate the effects of obesity in Mexico. Further, a pilot study in Mexican adults showed promising results [20]. Thus, to determine whether digitally delivered ILMs reduce weight loss in Mexican adults, the present study evaluated the efficacy of a web‐based ILM protocol adapted from the DPP (DPP‐web) on weight loss in Mexican adults with overweight or obesity at three and 6 months compared to a wait‐list control group.

## 2 Methods

To estimate the effect of the DPP‐web, a randomized controlled trial with parallel groups and 1:1 allocation ratio was conducted, considering a wait‐list control group.

The primary outcome was the mean change in body weight from baseline to 3 months post‐baseline between the two groups. Secondary outcomes were differences in biochemical parameters (fasting glucose, total cholesterol, LDL cholesterol, HDL cholesterol, triglycerides, and gamma glutamyltransferase) from baseline to 3 months as well as mean changes from baseline to three and 6 months in body mass index, fat percentage, waist circumference, systolic and diastolic blood pressure, depressive symptoms, Health‐Related Quality of Life (HRQoL) and the number of participants achieving a weight loss greater than 5% of initial body weight. Change in body weight from baseline to 6 months was also evaluated as a secondary outcome.

The protocol was registered in Clinical Trials (NCT03629301) and approved by the Bioethics Committee of the Department of Medicine of the University of Sonora (DMCS/CBIDMCS/D‐110).

### 2.1 Population

Eligible participants were adults (≥ 18 and ≤ 60 years) with overweight or obesity (BMI ≥ 25 kg/m^2^ and ≤ 45 kg/m^2^), and residents of the city of Hermosillo, Sonora. In addition, requirements included having internet access at home, a device with a camera and internet connection, basic computer skills, an active Facebook account, and the ability to record food consumption for 5 days prior to baseline measurements.

Exclusion criteria included any clinical diagnoses or medications affecting body weight, conditions contraindicating weight loss or exercise, relatives in the study, weekly alcohol intake over 280g, ongoing weight loss treatments, psychiatric disorders impacting treatment adherence, recent weight loss exceeding 5%, systolic blood pressure above 160 mmHg, or upcoming residential moves.

Facebook ads were used for recruitment, targeting individuals who met the specific criteria. Videos were shared on our University Center's Facebook page, offering a free online weight loss program with immediate or delayed access. Interested individuals scheduled video interviews to confirm eligibility. To participate, a 5‐day food diary had to be submitted as a commitment indicator. No compensation was received by the participants, and there was no in‐person contact with the interventionist.

### 2.2 Measures

The study was conducted from April to October 2019, with measurements taken in a university clinic at three key time points within the intervention: baseline, three months, and six months. Within a 2‐week data collection window for each measurement time, all assessments were performed on the same day by two staff members blinded to the assignment group of the participants. Participants were instructed not to disclose their group allocation during the assessments.

Participants were required to adhere to specific pre‐assessment instructions, which included maintaining a minimum 12‐h fasting period, wearing lightweight clothing, refraining from physical activity, and abstaining from alcohol consumption within the preceding 24 h. A blood sample from the antecubital veins was collected by an experienced phlebotomist between 7:00 and 9:00 a.m. Subsequently, a trained dietitian conducted a brief interview to collect demographic characteristics, such as age, sex, education level, marital status, and monthly income, measured height, body weight, waist circumference, fat percentage, systolic and diastolic blood pressure, applied the surveys for measuring depressive symptoms and HRQoL, and enrolled participants in the intervention.

Venous blood samples were collected using both EDTA and non‐anticoagulant tubes, using a standard Vacutainer method (Becton Dickinson, Franklin Lakes, NJ, USA). Plasma and serum samples were further used to measure fasting plasma glucose, gamma‐glutamyl transferase, triglycerides, total cholesterol, LDL cholesterol, and HDL cholesterol using standardized commercially available diagnostic tests in a Hitachi Modular P800 Analyzer (Roche Diagnostics Co., Indianapolis, IN, USA).

Weight and fat percentage were measured with a digital scale and a body fat monitor (medical Body Composition Analyzer, Seca Gmbh & Co. Kg Hammer Steindamm 9–25) and height with a Seca brand stadiometer (model 284, Seca Gmbh & Co. Hammer Steindamm 9–25. Germany: measuring capacity 30–220 cm). Waist circumference was measured at the level of the umbilical scar, using an anthropometric tape (Lufkin Executive Thinline with a scale from 0 to 200 cm. Model: W606PMMX). The blood pressure measurement was conducted using an Omron brand manometer (model HEM‐907XL, Omron Healthcare Co. Ltd, USA) following the American Heart Association recommendations [21].

The 36‐Item Short Form Health Survey (SF‐36) was used to measure changes in HRQoL [22]. This questionnaire includes 36 items that assess 8 domains: physical functioning, role limitations due to physical problems, bodily pain, general health perceptions, vitality, social functioning, role limitations due to emotional problems, and mental health. Each domain receives a score from 0 to 100, where a higher score indicates a better‐perceived health state. The internal consistency of the SF‐36 has been validated in Mexico with a convergent validity coefficient of *r* = 0.40, and a Cronbach's alpha reliability coefficient range between 0.70 and 0.84 for most items, with 0.56 for the emotional role [22, 23].

The Beck Depression Inventory‐II (BDI‐II), a 21‐item self‐report questionnaire, was used to assess the severity of depressive symptoms [24]. The scale's items align with the diagnostic criteria for a major depressive episode as defined by the American Psychiatric Association, assessing various aspects of mood, cognitive, and somatic symptoms. Each item is scored on a scale of 0–3, yielding a total score from 0 to 63, where a higher score represents a higher symptom severity. The instrument's internal consistency has been validated in the Mexican population, with Cronbach's alpha reliability coefficients reported to range from 0.87 to 0.92 [25]. Additionally, strong concurrent validity has been demonstrated with a coefficient of 0.87 when compared to the Zung scale [25].

### 2.3 Randomization

After baseline measurements, the statistician, not involved in recruitment or measurements, received the database with only age, weight, height, BMI, and gender. Participants were randomly assigned by sex‐stratified blocks in a 1:1 ratio using Research Randomizer 4.0 [26]. Offsite randomization at a single time point, without personal details of the participants, ensured allocation concealment.

### 2.4 Statistical Analysis

The sample size was estimated based on a pilot study conducted by the same research team [20]. A mean difference in weight change between groups of 3.75 kg and a standard deviation of 3.90 kg was found. Considering two groups, a power of 80%, a *p* value of 0.05 and a 30% dropout rate, the estimated sample size needed was 49 participants; however, a target of 62 participants was established.

All analyses were performed using STATA SE/14.2 [27] considering a *p* value of < 0.05 as the threshold for statistical significance. Normality was evaluated graphically for all variables. To assess differences between groups at baseline and the treatment effect (change from baseline) of all outcomes at three and 6 months, a two tailed t‐test for independent samples was conducted as established in the protocol. Additionally, a mixed‐effects analysis using the xtmixed command was performed. This analysis utilized a repeated measures design, treating individual subjects as random effects to account for the repeated measurements within the same individuals. The assumption for this analysis was that the random effects followed a multivariate normal distribution centered at zero with an unstructured variance‐covariance matrix, which allowed the exploration of the influence of both treatment group and time on changes in weight, adjusted for the variables that showed differences between groups at baseline and other variables that potentially could have an impact. The chi‐squared test was used to compare proportions.

All analyses followed the intention‐to‐treat principle, considering the Markov Chain Monte Carlo method for multiple imputations [28]. Although multiple imputations were not initially planned, they were implemented to adhere to best practices and ensure the robustness of the analysis in the presence of missing data, with 20 imputations performed for 14 missing values at 3 months an 40 imputations to estimate 25 missing values at 6 months. For all outcomes, baseline values were used as adjusting variables. Additionally, when a significant correlation (*r* > 0.4 and *p* < 0.05) was observed between the number of attended sessions and values at the measured time points, this was incorporated as an additional adjusting variable. To pool the results from the imputed datasets, we followed the process recommended by Rubin [29]. Finally, a sensitivity analysis was performed to assess differences in baseline characteristics and outcome measurements between datasets containing imputed values and those with complete data (Supporting Information S1: Table S1). No outcome data were excluded.

### 2.5 Interventions

#### 2.5.1 Intervention: DPP‐Web

The 6‐month intervention comprised two main components: 18 educational sessions (ES) and 18 nutrition counseling sessions (NCS). The first 12 ESs and 12 NCSs were delivered weekly in the first 3 months, with the remaining sessions in the 3‐month follow‐up phase delivered every two weeks. Both the ES and the NCS were implemented by a dietitian who had undergone a year of specialized training in implementing the DPP in‐person and had previously conducted a 3‐month online adaptation tailored for the Mexican population. During the current study, the dietitian was pursuing a PhD degree.

A web‐based app was developed by the Information Technology department at the University of Sonora. This platform allowed participants to access ES, schedule their NCS, upload a weekly food diary, log their anthropometric measurements and complete a questionnaire for each session. Neither the weekly self‐reported measurements nor the food diary were employed as evaluation criteria for the efficacy of the intervention. The questionnaires included activities aligned with the content of the ES and were used to gauge the uptake of each ES.

The program included 18 sessions: 16 adapted from the 2017 Lifestyle Group Balance DPP and two additional sessions rooted in the Mexican Equivalent Food System [10, 30]. The adaptation process focused on both content and format. Content adaptations included translating English handouts to Spanish [10], redesigning materials with culturally appropriate images, optimizing fonts for screen readability, and adding sessions based on the Mexican Equivalent Food System [30]. For format adaptations, PDFs akin to the original version were created for the handbook. However, reinforcement questionnaires were digitalized using Google Forms, and for each, a 10–15‐min pre‐recorded video that summarized key learning objectives was created. All study materials in PDF are available in Spanish and can be freely accessed [31].

The ESs were designed to promote self‐directed learning and user‐friendliness. In each session, the corresponding pre‐recorded video was uploaded weekly (or every 2 weeks in the 3‐month follow‐up phase) to the web‐based platform, along with the adapted PDF materials and a Google Form questionnaire. This approach allowed participants the flexibility to access these resources at their convenience. To further enrich participant engagement and support, a Facebook group moderated by the interventionist was established. Within this virtual group setting, the content of each ES session was not only reinforced but also actively discussed, fostering a collaborative learning environment.

All NCS were exclusively conducted via the Facebook Messenger video call service, with no in‐person meetings. In the initial NCS session, which lasted approximately 60 min, participants set their weight loss goals, typically targeting a 5%–10% reduction. During this session, comprehensive guidance on self‐weighing techniques and accurate measurement of waist circumference was provided.

Subsequent NCS sessions, with durations ranging from 30 to 40 min, involved discussions centered around their food diaries, addressing inquiries related to the ES, providing individualized feedback, conducting weekly assessments grounded in self‐reported anthropometric measurements, and reinforcing the cognitive behavioral strategies introduced in the ES. These strategies encompass goal setting, acquiring fundamental nutrition knowledge, honing self‐regulation and self‐efficacy skills, and navigating the process of identifying and reframing intrusive thoughts related to nutrition, all of which were fine‐tuned to specifically address dietary aspects.

The dietary treatment was modeled after the Look AHEAD study [32, 33], which used a calorie restriction that depended on initial body weight. For people who weighed less than 114 kg, the calorie goal was 1200–1500 kcal per day and for people who weighed more than 114 kg, the goal was 1500–1800 kcal per day [32]. The macronutrient distribution was based on the Dietary Reference Intakes suggested by the Institute of Medicine [34]. Specifically, all meal plans were designed to adhere to the recommended ranges of 45%–65% of total energy from carbohydrates, 20%–35% from protein, and 10%–35% from fat [34].

For the meal plans, a dietitian created 8 weekly menus considering local food preferences and the menus were adapted to 1200, 1400, 1600, and 1800 calories. For each calorie target, different options of meal structures with calorie‐equivalent meals were offered to the participants. These options included the use of optional meal replacements and optional snacks between meals. Notably, no commercial meal replacements were consumed; instead, participants who chose this option opted for homemade alternatives (smoothies consisting of milk, fruit, nuts or seeds, and psyllium).

In the initial 8 weeks, participants were provided with healthy structured meal plans, considering their preferences and calorie targets. Starting from week nine and continuing until the end of the intervention, participants were encouraged to plan their own meals, enabling them to sustain their progress towards achieving their weight loss goals.

### 2.6 Wait‐List

The participants allocated to the wait‐list control group were offered an ILM program for weight loss either face‐to‐face or online at the end of the 6‐month study. In addition, they received an electronic brochure at the beginning of the study with educational material based on the clinical practice guidelines for the diagnosis and treatment of overweight and exogenous obesity [35].

## 3 Results

**FIGURE 1:**
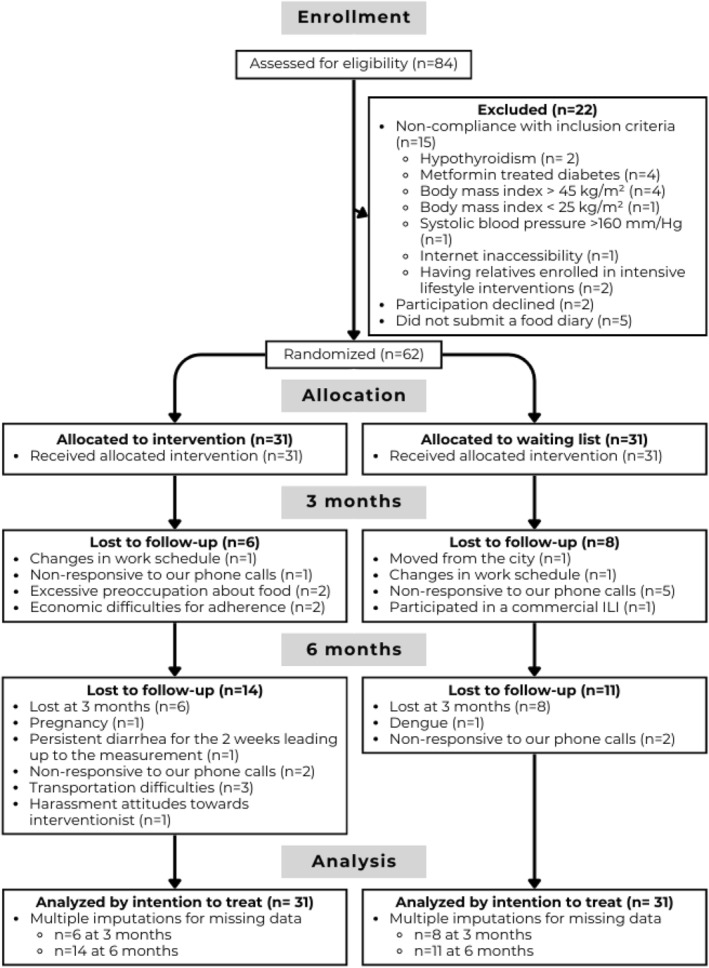
Diagram of the flow of the 62 participants according to the consolidated standards for reporting of trials.

Of 84 potential participants assessed for eligibility, a total of 62 participants were randomized to either the DPP‐web group (*n* = 31) or wait‐list group (*n* = 31) following a 1:1 allocation ratio (Figure 1). Within the DPP‐web group, six participants dropped out at 3 months and another eight at 6 months. Within the wait‐list group, eight participants dropped out at 3 months and another three at 6 months. Outcome assessment was completed for 48 out of 62 of the randomized participants at three months (77%) and for 37 out of 62 at six months (60%). No harms were reported.

### 3.1 Baseline Characteristics

The mean age of the participants was 31.5 (SD 8.6) years, the mean BMI 33.4 (SD 4.9) kg/m^2^, and 50 out of 62 were women (81%). The groups did not differ significantly on any demographic or anthropometric variables at baseline (Table 1). The DPP‐web group reported higher scores than the wait‐list group in the bodily pain and health transition subscales of the HRQoL questionnaire (*p* < 0.01 and *p* = 0.02, respectively), which indicates reduced perceived pain levels and improved perceived health compared to the preceding 3 months. These variables were subsequently incorporated into the mixed‐effects analysis to assess if they altered the results.

**TABLE 1:** Baseline characteristics of the enrolled participants according to their allocation group (*n* = 62).

Outcome	DPP‐web (*n* = 31)	Control (*n* = 31)	*p* ^a^
Sex, *n* (%)			
Female	25 (81)	25 (81)	0.99
Male	6 (19)	6 (19)	
Age (years), mean (SD)	32.77 (8.55)	30.29 (8.61)	0.26
Education level, *n* (%)			
Basic education	1 (3)	5 (16)	0.09
High school degree	7 (23)	12 (39)	
Bachelors degree	21 (68)	12 (39)	
Postgraduate degree	2 (6)	2 (6)	
Marital status, *n* (%)			
Single or divorced	20 (65)	16 (52)	0.30
Married or cohabitation	11 (35)	15 (48)	
Monthly income (MXN), *n* (%)			
less than 10,000	17 (55)	22 (71)	0.20
10,000 or more	14 (45)	9 (29)	
Weight (kg), mean (SD)	89.85 (17.58)	92.26 (16.01)	0.57
Body fat (%), mean (SD)	44.14 (4.92)	43.37 (6.05)	0.58
BMI (kg/m^2^), mean (SD)	32.96 (5.30)	33.78 (4.40)	0.51
Waist circumference (cm), mean (SD)	103.39 (11.26)	105.79 (10.25)	0.38
SBP (mm/Hg), mean (SD)	113.03 (14.08)	111.42 (11.57)	0.62
DBP (mm/Hg), mean (SD)	74.32 (10.86)	69.77 (6.68)	0.05
Depressive symptoms: BDI, mean (SD)	13.00 (8.18)	16.32 (9.84)	0.15
HRQoL:SF‐36^b^, mean (SD)			
Physical functioning	80.65 (15.80)	79.52 (20.10)	0.81
Role limitations due to physical problems	68.55 (34.14)	55.00 (42.75)	0.18
Bodily pain	77.19 (18.79)	58.47 (27.12)	**< 0.01**
Social functioning	68.95 (22.33)	57.26 (25.98)	0.06
Mental health	59.87 (18.11)	57.16 (15.97)	0.53
Role limitations due to emotional problems	45.15 (29.26)	43.01 (30.07)	0.78
Vitality	47.58 (17.36)	43.87 (17.64)	0.41
General health perceptions	56.77 (16.56)	50.00 (17.75)	0.13
Health transition	54.03 (25.08)	40.32 (20.08)	**0.02**
Biochemical variables^c^, mean (SD)			
Fasting glucose	82.10 (7.83)	82.83 (8.94)	0.73
Total cholesterol	168.00 (34.14)	169.27 (35.40)	0.89
Tryglicerides	110.26 (52.54)	118.00 (104.54)	0.72
High density lipoprotein	40.58 (9.88)	39.70 (8.40)	0.71
Low density lipoprotein	109.45 (35.89)	107.90 (36.02)	0.87
Gamma glutamil aminotransferase	19.32 (13.97)	15.80 (8.18)	0.23

*Note:* Boldface indicates statistical significance (*p* < 0.05).

Abbreviations: BDI, Beck depression inventory; BMI, body mass index, calculated as weight in kilograms divided by height in meters squared; DBP, diastolic blood pressure; HRQoL, health‐related quality of life; SBP, systolic blood pressure.

^a^ For comparing baseline values between groups, chi‐square test was used for categorical data and a two tailed *t*‐test for independent samples was used for continuous data.

^b^ A higher score indicates a better health state.

^c^ Wait‐list control (*n* = 30) and DPP‐web (*n* = 31).

### 3.2 Attendance at Intervention Activities

On average, participants from the intervention group attended 12 out of 18 NCS (67%), answered seven out of 18 questionnaires about the ES (33%), and completed their food diary 11 out of 24 weeks (46%). The participants who attended the final measurement appointment at six months had better attendance in the DPP‐web, having attended on average 14 out of 18 (78%) NCS, answered 8 out of 18 (44%) questionnaires about the ES, and completed their food diary 17 out of 24 weeks (65%).

### 3.3 Change in Body Weight

**FIGURE 2:**
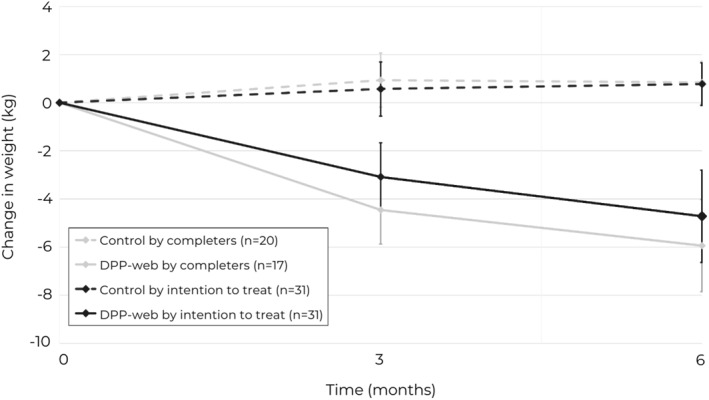
Mean change in weight (95% CI) for the participants of the DPP‐web group and the control group at three and 6 months, according to the intention‐to‐treat analysis in which multiple imputations were used to estimate missing values (black colored lines) or the completers analysis that only includes the data of participants who attended the measurements at 6 months (grey colored lines).

In the intention‐to‐treat analyses, significant differences were observed between groups at both 3 months (−3.65 kg, 95% CI −5.41, −1.89, *p* < 0.01) and 6 months (−5.50 kg, 95% CI −7.58, −3.41, *p* < 0.01). The mixed‐effects analysis further confirmed the statistical significance of the DPP‐web and time interaction for both the three‐ and 6‐month measures (*p* < 0.01, log likelihood = −455.23) (Figure 2 and Supporting Information S1: Table S2).

While baseline pain perception showed a significant negative association with change in weight at 6 months, the impact of the DPP‐web on weight changes remained significant, even when accounting for baseline pain perception as a confounder (*p* < 0.01). The mixed‐effects analysis showed that for every unit increase in the baseline pain perception score, indicating lower perceived pain, there is a corresponding reduction of 0.04 kg (95% CI −0.07, −0.02, *p* < 0.01) in weight change. In contrast, perceiving an improvement in health did not yield a significant effect on weight change (*p* = 0.80) (Supporting Information S1: Table S2).

For the completer analysis (Supporting Information S1: Table S3), the difference between groups was −4.73 kg (95% CI −6.65, −2.80, *p* < 0.01) at 3 months (*n* = 48) and −6.78 kg (95% CI −10.28, −3.28, *p* < 0.01) at 6 months (*n* = 37). The mixed‐effects analysis with completers showed the same trend as with the intention‐to‐treat analysis (*p* < 0.01) (Supporting Information S1: Table S2).

## 4 Change in Secondary Outcomes

Compared to the wait‐list control group, participants in the DPP‐web group achieved greater reductions in BMI, waist circumference, and body fat at three and 6 months according to both the intention‐to‐treat and completers analyses (Table 2 and Supporting Information S1: Table S3).

**TABLE 2:** Changes in body weight and other variables at three and 6 months of intervention by intention‐to‐treat analysis^a^ (*n* = 62).

Outcome	DPP‐web at 3 months^b^ (*n* = 31)	Control at 3 months^b^ (*n* = 31)	Treatment effect at 3 months^c^	DPP‐web at 6 months^b^ (*n* = 31)	Control at 6 months^b^ (*n* = 31)	Treatment effect at 6 months^c^
	Mean (SD)	Mean (SD)	Mean (95% CI); *p* value	Mean (SD)	Mean (SD)	Mean (95% CI); *p* value
Body weight (kg)	−3.08 (3.80)^e^	0.57 (3.09)	−3.65 (−5.41, −1.89); **≤ 0.01**	−4.72 (5.22)^e^	0.78 (2.41)	−5.50 (−7.58, −3.41); **≤ 0.01**
Body weight (%)	−3.18 (3.86)^e^	0.84 (3.07)	−4.02 (−5.80, −2.25); **≤ 0.01**	−4.88 (4.95)^e^	0.87 (2.82)	−5.75 (−7.81, −3.70); **≤ 0.01**
Body fat (%)	−1.40 (1.79)^e^	−0.02 (1.34)	−1.38 (−2.19, −0.58); **≤ 0.01**	−2.38 (1.97)^e^	−0.13 (1.21)	−2.25 (−3.08, −1.42); **≤ 0.01**
Body mass index (kg/m^2^)	−1.08 (1.30)^e^	0.26 (1.04)	−1.35 (−1.94, −0.75); **≤ 0.01**	−1.67 (1.74)^e^	0.29 (0.87)	−1.96 (−2.66, −1.25); **≤ 0.01**
Waist circumference (cm)	−4.05 (4.02)^e^	−0.52 (3.20)	−3.53 (−5.38, −1.69); **≤ 0.01**	−6.02 (4.70)^e^	−0.72 (3.04)	−5.30 (−7.31, −3.29); **≤ 0.01**
Systolic blood pressure (mm/Hg)	0.47 (9.94)	2.22 (9.80)	−1.75 (−6.76, 3.27); 0.49	−2.67 (9.18)	−0.27 (9.20)	−2.39 (−7.06, 2.28); 0.31
Diastolic blood pressure (mm/Hg)	−3.06 (6.26)^e^	0.57 (5.72)	−3.64 (−6.68, −0.59); **0.02**	−2.43 (6.84)	0.10 (8.50)	−2.53 (−6.45, 1.39); 0.20
Depressive symptoms: BDI (score)	−5.32 (5.47)^e^	−0.31 (5.55)	−5.01 (−7.82, −2.21); **≤ 0.01**	−5.98 (8.80)^e^	−2.76 (11.26)	−3.22 (−8.36, 1.91); 0.21
HRQoL:SF‐36 (score)						
Physical functioning	7.92 (9.44)^e^	1.53 (11.45)	6.39 (1.06, 11.72); **0.02**	5.95 (14.13)^e^	2.20 (11.94)	3.75 (−2.90, 10.39); 0.26
Role limitations due to physical problems	14.83 (23.49)^e^	3.42 (37.46)	11.41 (−4.55, 27.37); 0.16	12.73 (23.48)^e^	10.90 (41.50)	1.82 (−15.63, 19.27); 0.83
Bodily pain	5.45 (20.50)	7.74 (15.19)^e^	−2.28 (−11.45, 6.88); 0.62	5.62 (12.47)^e^	9.12 (18.97)^e^	−3.50 (−11.67, 4.68); 0.39
Social functioning	6.97 (26.34)	6.59 (22.92)	0.38 (−12.16, 12.92); 0.95	9.84 (21.30)^e^	6.52 (24.36)	3.32 (−8.31, 14.94); 0.57
Mental health	5.19 (17.48)	2.99 (12.95)	2.20 (−5.62, 10.01); 0.58	5.63 (15.05)	6.78 (14.05)	−1.15 (−8.54, 6.25); 0.76
Role limitations due to emotional problems	10.17 (25.84)^e^	−0.45 (30.57)	10.61 (−3.77, 24.99); 0.15	15.16 (40.13)^e^	12.16 (30.30)	3.00 (−15.06, 21.07); 0.74
Vitality	11.09 (16.17)^e^	6.31 (15.45)	4.78 (−3.26, 12.81); 0.24	9.84 (15.25)^e^	7.04 (15.26)	2.80 (−4.95, 10.55); 0.47
General health perceptions	12.83 (16.57)^e^	6.59 (14.45)^e^	6.24 (−1.66, 14.13); 0.12	13.68 (12.13)^e^	7.13 (13.75)	6.55 (−0.04, 13.14); 0.05
Health transition	22.72 (25.55)^e^	13.75 (23.11)^e^	8.97 (−3.41, 21.35); 0.15	20.24 (23.10)^e^	11.05 (15.87)^e^	9.18 (−0.91, 19.27); 0.07
Biochemical variables^d^						
Fasting glucose (mg/dL)	−1.49 (12.22)	−3.72 (9.80)	2.24 (−3.45, 7.92); 0.43			
Total cholesterol (mg/dL)	4.41 (26.08)	3.05 (27.41)	1.36 (−12.34, 15.07); 0.84			
Tryglicerides (mg/dL)	−17.64 (43.18)	−27.81 (64.61)^e^	10.17 (−17.90, 38.24); 0.47			
High density lipoprotein (mg/dL)	0.04 (6.76)	0.25 (7.13)	−0.20 (−3.76, 3.36); 0.91			
Low density lipoprotein (mg/dL)	−1.70 (30.75)	−2.33 (31.95)	0.63 (−15.43, 16.69); 0.94			
Gamma glutamyltransferase (mg/dL)	0.41 (10.01)	2.77 (5.73)^e^	−2.35 (−6.54, 1.83); 0.26			

*Note:* Boldface indicates statistical significance (*p* < 0.05).

Abbreviations: BDI, Beck depression inventory; BMI, body mass index, calculated as weight in kilograms divided by height in meters squared; DBP, diastolic blood pressure; HRQoL, health‐related quality of life; SBP, systolic blood pressure; SF‐36, 36‐item short form health survey.

^a^ All analyses followed the intention‐to‐treat principle using the Markov Chain Monte Carlo method for multiple imputations to estimate missing values (14 missing values at three months and 25 at six months).

^b^ The change at 3 and 6 months for each participant was calculated as the final value minus the baseline value.

^c^ Treatment effect is defined as the change in the DPP‐web group minus the change in the wait‐waiting list control group. Data are presented as means (95% confidence intervals); *p* value. Groups were compared using a two tailed *t*‐test for independent samples. A positive value in the following variables represents an improvement: subscales of the HRQoL questionnaire (SF‐36) and in high‐density lipoprotein. In all other cases, a negative value represents an improvement.

^d^ Wait‐list control (*n* = 30) and DPP‐web (*n* = 31).

^e^ Statistical significance (*p* ≤ 0.05) within the group assessing differences using paired t‐tests.

Multiple imputation analyses: The weight change at 3 months was −3.08 (95% CI −4.48, −1.69) kg for the intervention group and 0.57 (95% CI −0.57, 1.70) kg for the control group, and the difference between groups was −3.65 (95% CI −5.41, −1.89) kg. The weight change at 6 months was −4.72 (95% CI −6.64, −2.80) for the intervention group and 0.78 (95% CI −0.11, 1.66) for the control group, and the difference between groups was −5.50 (95% CI −7.58, −3.41). Completer analyses: The weight change at 3 months was −4.46 (95% CI −6.55, −2.36) kg for the intervention group and 0.94 (95% CI −0.36, 2.24) kg for the control group, and the difference between groups was −5.39 (95% CI −7.69, −3.10) kg. The weight change at 6 months was −5.94 (95% CI −9.23, −2.65) for the intervention group and 0.84 (95% CI −0.55, 2.23) for the control group, and the difference between groups was −6.78 (95% CI −10.28, −3.28).

At 3 months, diastolic blood pressure, depressive symptoms, and physical functioning (HRQoL) improved but were not sustained at 6 months. No significant differences were found in systolic blood pressure, other HRQoL scores, or biochemical variables (Table 2). At 6 months, 35% of participants achieved > 5% weight loss according to the intention‐to‐treat analysis, while 41% did so in the completer analysis. In both cases, no participants in the control group achieved this goal (*p* < 0.01, Supporting Information S1: Table S4).

## 5 Discussion

This study found that the DPP‐web led to significant weight loss among adults with overweight or obesity, along with improvements in BMI, waist size, and body fat percentage. At 3 months, participants reported feeling physically better and less depressed, along with a decrease in diastolic blood pressure. However, these improvements were not sustained at 6 months, and no significant changes were observed in systolic blood pressure, other HRQoL scores, or biochemical variables at either the three‐ or 6‐month follow‐up. This study is believed to be the first randomized controlled trial of an online ILM program for weight loss in Latin America, besides the pilot study conducted by the research team.

The results at 3 months of −3.65 kg analyzed by intention‐to‐treat and −4.73 kg considering only completers, closely mirrored those obtained in the pilot study with the same intervention, which showed a difference of −3.75 kg (95% CI −6.67, −0.84) by intention‐to‐treat analysis and −4.69 kg (95% CI −8.29, −1.09) by completers analysis, compared to the wait‐list group [20]. Additionally, the findings at 6 months, analyzed by intention‐to‐treat, showed a difference of −5.50 kg, which aligns with outcomes from face‐to‐face interventions across diverse real‐world clinic settings where participants typically lost between 2 and 6 kg in the same timeframe [12]. Furthermore, a meta‐analysis conducted by Bian et al. reported a weight loss of 4.81 kg (95% CI 3.9–5.7) in the intervention arm of an online ILM protocol adapted from the DPP over a range of three to 15 months [17] This result closely resembled the weight loss achieved in the intervention group of the present study after 6 months, which was 4.72 kg.

The retention rates at 6 months (77% and 60%) were similar to those reported in a systematic review of internet‐based interventions for weight loss conducted by Sherrington et al., where between 79% and 88% of participants completed the first 3 months of the intervention and between 57.1% and 89.5% completed the first 6 months [36].

Although there is a significant body of evidence showing the benefits of ILMs, including digital versions, for the treatment of obesity and associated comorbidities [3, 36], these interventions are currently unavailable to the Mexican population through the public health system [19]. Data at the national level show that 80% of Mexicans with obesity are not diagnosed and 92% do not receive any treatment [37]. The few patients who do receive an intervention with infrequent appointments (one session every one to 4 months) that does not affect body weight [19]. At the time this study was conducted, ILM was not included in the Mexican Standard for the comprehensive treatment of overweight and obesity [38]. While the updated guidelines now incorporate lifestyle recommendations [39], they still lack a standardized definition of ILM protocols and clear guidelines for their implementation [40], with few trained professionals and limited availability. In contrast, in the United States, ILM protocols are considered the gold standard for obesity treatment and are covered by some state health insurance programs, often as part of national iniciatives [41]. Ideally, Mexico should follow this model to ensure broader access to evidence‐based obesity care.

The study has several important limitations. The online format introduced potential for variability in program implementation, as the lack of direct supervision may have led to inconsistent application of the recommendations. Furthermore, adherence to the diet and physical activity protocols was not tracked, and there was no long‐term follow‐up to determine if the observed effects could be sustained over time. While some psychological factors that may influence adherence to the program, such as depression and health related quality of life, were measured as part of the study, they were not addressed as part of the ILM program. Additionally, the study was conducted exclusively in a single region of Mexico. Given the cultural variations across Latin American countries and within different states of Mexico, the generalizability of our findings to other regions may be limited.

This is the first randomized controlled trial of an online ILM protocol for weight loss in Latin America, besides the pilot study conducted by the same research team. The results obtained in the present study were achieved using high‐quality methods, and in the context of an intervention that was delivered 100% online.

## 6 Conclusion

The DPP‐web significantly reduced the body weight of Mexican adults with overweight or obesity at 3 months, and its effect was maintained at 6 months, representing an effective and accessible strategy for obesity treatment. Even though it has a lower treatment effect than face‐to‐face interventions, it is likely to be more affordable and has a greater potential reach than face‐to‐face interventions. Policymakers in Mexico and other low‐ and middle‐income countries should explore options to expand access to both online and in‐person ILM protocols for weight loss among adults with overweight. Spanish‐speaking health professionals are encouraged to utilize the available and customizable materials from this study to implement the intervention effectively.

## Author Contributions

R.G.D.Z. and A.L.R.Y. conceived and designed the study. A.L.R.Y. conducted the intervention implementation. T.J.M.C. measured all anthropometrical data and A.L.M.P. all the biochemical analysis measurements. A.L.M.P. and M.C.C.P. analyzed the biochemical results. R.G.D.Z., J.E.R., and A.O.V. provided statistical and clinical trial design expertise, and M.M.H., T.J.M.C., A.L.M.P., and M.C.C.P. contributed ideas for the study's design. All authors contributed to the refinement of the study protocol and read and approved the final manuscript.

## Funding

This work received support from the Department of Chemical‐Biological Sciences and the Faculty of Biological and Health Sciences at the University of Sonora. The support of CONAHCyT for a graduate scholarship [No. 492921] is gratefully acknowledged. Funding for the computer equipment and physical space for the measurements, as well as the biochemical analyses and nutritional assessment equipment, was provided by the University of Sonora. The study's design, management, analysis, and reporting are entirely independent of the University of Sonora.

## Conflicts of Interest

The authors declare no conflicts of interest.

## Supporting information


Supporting Information S1

## References

[1] H. Dai , T. A. Alsalhe , N. Chalghaf , M. Ricco , N. L. Bragazzi , and J. Wu , “The Global Burden of Disease Attributable to High Body Mass Index in 195 Countries and Territories, 1990‐2017: An Analysis of the Global Burden of Disease Study,” PLoS Medicine 17, no. 7 (July 2020): e1003198, 10.1371/journal.pmed.1003198. 32722671 PMC7386577

[2] S. Barquera and M. White , “Treating Obesity Seriously in Mexico: Realizing, Much Too Late, Action Must be Immediate,” Obesity 26, no. 10 (October 2018): 1530–1531, 10.1002/oby.22296. 30277025

[3] T. A. Wadden , J. S. Tronieri , and M. L. Butryn , “Lifestyle Modification Approaches for the Treatment of Obesity in Adults,” American Psychologist 75, no. 2 (February–March 2020): 235–251, 10.1037/amp0000517. 32052997 PMC7027681

[4] The Diabetes Prevention Program Research Group . “The Diabetes Prevention Program (DPP): Description of Lifestyle Intervention,” Diabetes Care 25, no. 12 (December 2002): 2165–2171, 10.2337/diacare.25.12.2165. 12453955 PMC1282458

[5] R. B. Goldberg , T. J. Orchard , J. P. Crandall , et al., “Effects of Long‐Term Metformin and Lifestyle Interventions on Cardiovascular Events in the Diabetes Prevention Program and Its Outcome Study,” Circ 145, no. 22 (2022): 1632–1641, 10.1161/CIRCULATIONAHA.121.056756. PMC9179081 35603600

[6] D. M. Nathan , P. H. Bennett , J. P. Crandall , et al., “Does Diabetes Prevention Translate into Reduced long‐term Vascular Complications of Diabetes?,” Diabetologia 62, no. 8 (2019): 1319–1328, 10.1007/s00125-019-4928-8. 31270584 PMC6818092

[7] Q. Gong , P. Zhang , J. Wang , et al., “Morbidity and Mortality After Lifestyle Intervention for People With Impaired Glucose Tolerance: 30‐Year Results of the Da Qing Diabetes Prevention Outcome Study,” Lancet Diabetes and Endocrinology 7, no. 6 (2019): 452–461, 10.1016/S2213-8587(19)30093-2. 31036503 PMC8172050

[8] D. A. Williamson , “Fifty Years of Behavioral/Lifestyle Interventions for Overweight and Obesity: Where Have we Been and Where Are we Going?,” Obesity 25, no. 11 (2017): 1867–1875, 10.1002/oby.21914. 28944593

[9] W. C. Knowler , E. Barrett‐Connor , S. E. Fowler , et al., “Reduction in the Incidence of Type 2 Diabetes With Lifestyle Intervention or Metformin,” New England Journal of Medicine 346, no. 6 (2002): 393–403, 10.1056/NEJMoa012512. 11832527 PMC1370926

[10] The Diabetes Prevention Program Research Group . Diabetes Prevention Program Group Lifestyle Balance™ Materials, (2021): Updated 2017. Accessed, August 25, 2021, https://web.archive.org/web/20210826030906/https://www.diabetesprevention.pitt.edu/group‐lifestyle‐balance‐materials/.

[11] R. Whittemore , “A Systematic Review of the Translational Research on the Diabetes Prevention Program,” Translational Behavioral Medicine 1, no. 3 (2011): 480–491, 10.1007/s13142-011-0062-y. 24073067 PMC3717627

[12] B. Armenta‐Guirado , T. Martinez‐Contreras , M. C. Candia‐Plata , et al., “Effectiveness of the Diabetes Prevention Program for Obesity Treatment in Real World Clinical Practice in a Middle‐Income Country in Latin America,” Nutrients 11, no. 10 (2019): 2324, 10.3390/nu11102324. 31581478 PMC6835923

[13] B. I. Armenta‐Guirado , R. G. Diaz‐Zavala , M. E. Valencia‐Juillerat , and T. Quizan‐Plata , “Obesity Management in the Primary Care Setting by an Intensive Lifestyle Intervention,” Nutricion Hospitalaria 32, no. 4 (2015): 1526–1534, 10.3305/nh.2015.32.4.9366. 26545513

[14] E. E. Mata‐González , C. Meza‐Peña , and C. Saldaña García , “Programas de Intervención a Través de Internet Para la reducción de peso en Adultos con Sobrepeso y Obesidad: Una Revisión Sistemática,” Revista Española de Nutrición Humana y Dietética 24, no. 4 (2020): 324–335, 10.14306/renhyd.24.4.984.

[15] K. L. Joiner , S. Nam , and R. Whittemore , “Lifestyle Interventions Based on the Diabetes Prevention Program Delivered via eHealth: A Systematic Review and meta‐analysis,” supplement, Preventive Medicine 100, no. Supplement C (2017): 194–207, 10.1016/j.ypmed.2017.04.033. 28456513 PMC5699208

[16] I. R. Podina and L. A. Fodor , “Critical Review and Meta‐Analysis of Multicomponent Behavioral e‐health Interventions for Weight Loss,” Health Psychology 37, no. 6 (2018): 501–515, 10.1037/hea0000623. 29733617

[17] R. R. Bian , G. A. Piatt , A. Sen , et al., “The Effect of Technology‐Mediated Diabetes Prevention Interventions on Weight: A Meta‐Analysis,” Journal of Medical Internet Research 19, no. 3 (2017): e76, 10.2196/jmir.4709. 28347972 PMC5387112

[18] T. Bianchi , “Internet Usage in Mexico–Statistics & Facts,” (2023): Updated March 8, 2023, https://web.archive.org/web/20230730051844/https://www.statista.com/topics/3477/internet‐usage‐in‐mexico/#topicOverview.

[19] R. G. Díaz‐Zavala , M. D. C. Candia‐Plata , T. D. J. Martínez‐Contreras , and J. Esparza‐Romero , “Lifestyle Intervention for Obesity: A Call to Transform the Clinical Care Delivery System in Mexico,” Diabetes, Metabolic Syndrome and Obesity 12 (2019): 1841–1859, 10.2147/dmso.s208884. PMC6750852 31571959

[20] A. Ruelas , M. Haby , T. Martínez‐Contreras , J. Esparza‐Romero , MdC. Candia Plata , and R. Díaz‐Zavala , Efficacy of a Web‐based Adaptation of the Diabetes Prevention Program with Online Nutritional Counselling for Weight Loss During the Holiday Season Among Adults with Overweight or Obesity: A Pilot Randomised Controlled Trial (Preprint), (2020).

[21] P. K. Whelton , R. M. Carey , W. S. Aronow , et al., “2017 ACC/AHA/AAPA/ABC/ACPM/AGS/APhA/ASH/ASPC/NMA/PCNA Guideline for the Prevention, Detection, Evaluation, and Management of High Blood Pressure in Adults: Executive Summary,” Hypertension 71, no. 6 (2018): 1269–1324, 10.1161/hyp.0000000000000066. 29133354

[22] A. L. Stewart , Measuring Functioning and well‐being: The Medical Outcomes Study Approach, 1st ed. (Duke University Press, 1992).

[23] M. A. Zúniga , G. T. Carrillo‐Jiménez , P. J. Fos , B. Gandek , and M. R. Medina‐Moreno , “Evaluación del estado de salud con La encuesta SF‐36: Resultados preliminares en México,” Salud Publica de Mexico 41, no. 2 (1999): 110–118, 10.1590/s0036-36341999000200005. 10343514

[24] A. T. S. Beck , A. Robert , and G. Brown , Beck Depression Inventory–II (BDI‐II) (APA PsycTests, 1996), 10.1037/t00742-000.

[25] S. Lima‐Quezada Agc and S. Galán Cuevas , “Scales and Instruments to Measure Depression in Primary Care,” Mexican Journal of Medical Research ICSA 9, no. 17 (2021): 22–27, 10.29057/mjmr.v9i17.5586.

[26] Research Randomizer. (2013), http://www.randomizer.org/.

[27] Stata Statistical Software. StataCorp LLC; 2015.

[28] M. A. Elobeid , M. A. Padilla , T. McVie , et al., “Missing Data in Randomized Clinical Trials for Weight Loss: Scope of the Problem, State of the Field, and Performance of Statistical Methods,” PLoS One 4, no. 8 (August 2009): e6624, 10.1371/journal.pone.0006624. 19675667 PMC2720539

[29] D. B. Rubin , “Multiple Imputation After 18+ Years,” Journal of the American Statistical Association 91, no. 434 (1996): 473–489, 10.1080/01621459.1996.10476908.

[30] A. B. Pérez Lizaur , A. L. Castro Becerra , B. Palacios González , and I. Flores Galicia , SMAE: Sistema Mexicano de Alimentos Equivalentes, 4th ed. (FNS, 2014).

[31] Centro de Promoción de Salud Nutricional . Manual DPP Adaptado: Updated June 13, 2020. Accessed, February 22, 2023, https://web.archive.org/web/20230222030950/http://www.salud‐nutricional.com/manual_DPP_adaptado/.

[32] The Look AHEAD Research Group . “The Look AHEAD Study: A Description of the Lifestyle Intervention and the Evidence Supporting it,” Obesity 14, no. 5 (2006): 737–752, 10.1038/oby.2006.84. 16855180 PMC2613279

[33] R. R. Wing and Look AHEAD Research Group . “Does Lifestyle Intervention Improve Health of Adults With Overweight/Obesity and Type 2 Diabetes? Findings From the Look AHEAD Randomized Trial,” Obesity 29, no. 8 (2021): 1246–1258, PMID: 33988896, 10.1002/oby.23158. 33988896

[34] Institute of Medicine . Dietary Reference Intakes for Energy, Carbohydrate, Fiber, Fat, Fatty Acids, Cholesterol, Protein, and Amino Acids (National Academies Press, 2005).1358

[35] Guía de práctica clínica: diagnóstico y tratamiento del sobrepeso y obesidad exógena [Clinical practice guideline: diagnosis and treatment of overweight and exogenous obesity] (Instituto Mexicano del Seguro Social). 2018. 23883468

[36] A. Sherrington , J. J. Newham , R. Bell , A. Adamson , E. McColl , and V. Araujo‐Soares , “Systematic Review and meta‐analysis of Internet‐Delivered Interventions Providing Personalized Feedback for Weight Loss in Overweight and Obese Adults,” Obesity Reviews 17, no. 6 (2016): 541–551, 10.1111/obr.12396. 26948257 PMC4999041

[37] D. Pérez‐Salgado , J. Valdés Flores , I. Janssen , and L. Ortiz‐Hernández , “Diagnosis and Treatment of Obesity Among Mexican Adults,” Obesity Facts 5, no. 6 (2012): 937–946, 10.1159/000346325. 23296335

[38] NOM‐008‐SSA3‐2017 . Para el tratamiento integral del sobrepeso y la obesidad [For the comprehensive treatment of overweight and obesity] (Diario Oficial de la Federación, 2018).

[39] E. A. Chávez‐Manzanera , J. M. Vera‐Zertuche , M. Kaufer‐Horwitz , et al., “Mexican Clinical Practice Guidelines for Adult Overweight and Obesity Management,” Current Obesity Reports 13, no. 4 (October 2024): 643–666, 10.1007/s13679-024-00585-w. 39356455 PMC11522083

[40] Protocolo Nacional de Atención Médica (PRONAM): Sobrepeso y Obesidad (Consejo de Salubridad General) (2025).

[41] N. Jannah , J. Hild , C. Gallagher , and W. Dietz , “Coverage for Obesity Prevention and Treatment Services: Analysis of Medicaid and State Employee Health Insurance Programs,” Obesity 26, no. 12 (2018): 1834–1840, 10.1002/oby.22307. 30426721

