# Supplementary material for: Efficacy of an Online Weight Loss Program for Mexican Adults: Findings From a Randomized Controlled Trial
Source: Obes Sci Pract. 2026 Jun 19;12(3):e70165. doi: 10.1002/osp4.70165 (PMC13280466; doi:10.1002/osp4.70165)
Supplement: Supplementary file 1 — Supporting Information S1 [file OSP4-12-e70165-s001.docx]

**Table S1.** Comparison of Imputed and Measured Values at 3 and 6 Months

| **Outcome** | | **Imputed values at 3 months (n=12)** | **Measured values at 3 months (n=50)** | **P^a^** | **Imputed values at 6 months (n=25)** | **Measured values at 6 months (n=37)** | **P^a^** |
| --- | --- | --- | --- | --- | --- | --- | --- |
| **Sex, n (%)** | |  |  |  |  |  |  |
|  | Woman | 12 (100) | 38 (76) | 0.06 | 22 (88) | 28 (76) | 0.23 |
|  | Male | 0 (0) | 12 (24) |  | 3 (12) | 9 (24) |  |
| **Age (years), Mean (SD)** | | 30.25 (6.52) | 31.84 (9.05) | 0.57 | 30.44 (7.06) | 32.27 (9.52) | 0.42 |
| **Education level, n (%)** | |  |  |  |  |  |  |
|  | Basic education | 1 (8) | 5 (10) | 0.66 | 3 (12) | 3 (8) | 0.85 |
|  | High school degree | 5 (42) | 14 (28) |  | 7 (28) | 12 (32) |  |
|  | Bachelors degree | 6 (50) | 27 (54) |  | 14 (56) | 19 (51) |  |
|  | Postgraduate degree | 0 (0) | 4 (8) |  | 1 (4) | 3 (8) |  |
| **Marital status, n (%)** | |  |  |  |  |  |  |
|  | Single or divorced | 7 (58) | 29 (58) | 0.98 | 14 (56) | 22 (59) | 0.78 |
|  | Married or cohabitation | 5 (42) | 21 (42) |  | 11 (44) | 15 (41) |  |
| **Monthly income (MXN), n (%)** | |  |  |  |  |  |  |
|  | less than 10,000 | 10 (83) | 29 (58) | 0.10 | 17 (68) | 22 (59) | 0.50 |
|  | 10,000 or more | 2 (17) | 21 (42) |  | 8 (32) | 15 (41) |  |
| **Weight (kg), Mean (SD)** | | 88.93 (14.99) | 91.57 (17.21) | 0.63 | 87.33 (14.04) | 93.57 (18.06) | 0.15 |
| **Body Fat (%), Mean (SD)** | | 43.35 (5.89) | 45.44 (2.89) | 0.08 | 43.93 (4.84) | 43.63 (5.93) | 0.83 |
| **BMI (kg/m^2^), Mean (SD)** | | 33.40 (1.33) | 33.36 (4.95) | 0.98 | 32.35 (4.08) | 34.06 (5.25) | 0.18 |
| **Waist Circumference (cm), Mean (SD)** | | 105.25 (10.67) | 104.43 (10.87) | 0.81 | 102.60 (11.30) | 105.93 (10.30) | 0.23 |
| **SBP (mm/Hg), Mean (SD)** | | 109.33 (9.38) | 112.92 (13.49) | 0.38 | 109.44 (11.37) | 114.11 (13.52) | 0.16 |
| **DBP (mm/Hg), Mean (SD)** | | 71.33 (7.28) | 72.22 (9.70) | 0.77 | 71.48 (9.05) | 72.43 (9.46) | 0.69 |
| **Depressive symptoms: BDI, Mean (SD)** | | 20.83 (10.45) | 13.18(8.22) | **<0.01** | 18.32 (11.01) | 12.19 (6.69) | **0.02** |
| **HRQoL:SF-36**^b^**, Mean (SD)** | |  |  |  |  |  |  |
|  | Physical functioning | 70.00 (22.56) | 82.50 (15.98) | **0.03** | 77.80 (19.26) | 81.62 (17.08) | 0.42 |
|  | Role limitations due to physical problems | 35.42 (37.63) | 68.37 (36.72) | **<0.01** | 52.00 (41.41) | 68.75 (36.04) | 0.10 |
|  | Bodily pain | 56.5 (26.71) | 70.55 (24.04) | 0.08 | 64.12 (25.41) | 70.34 824.73) | 0.34 |
|  | **Social functioning** | **46.88 (30.21)** | **67.00 (21.83)** | **0.01** | 55.50 (27.74) | 68.24 (21.37) | 0.05 |
|  | Mental health | 52.67 (17.63) | 59.92 (16.71) | 0.19 | 52.64 (17.80) | 62.49 (15.42) | **0.02** |
|  | Role limitations due to emotional problems | 38.88 (27.84) | 45.33 (29.95) | 0.50 | 45.32 (30.26) | 43.24 (29.27) | 0.79 |
|  | Vitality | 40.42 (21.89) | 47.00 (16.23) | 0.24 | 43.60 (20.18) | 47.16 (15.48) | 0.44 |
|  | General health perceptions | 47.92 (12.52) | 54.70 (18.19) | 0.22 | 53.00 (18.54) | 53.65 (16.78) | 0.89 |
|  | Health transition | 39.58 (31.00) | 49.00 (21.40) | 0.22 | 46.00 (26.69) | 47.97 (21.55) | 0.75 |
| **Biochemical variables**^c^**, Mean (SD)** | |  |  |  |  |  |  |
|  | Fasting glucose | 82.75 (6.77) | 82.39 (8.73) | 0.89 | 81.36 (6.49) | 83.22 (9.42) | 0.40 |
|  | Total cholesterol | 170.67 (42.33) | 168.12 (32.77) | 0.82 | 165.56 (37.36) | 170.75 (32.71) | 0.57 |
|  | Triglycerides | 98.08 (54.29) | 117.98 (87.13) | 0.77 | 104.08 (80.40) | 121.00 (83.03) | 0.43 |
|  | High Density Lipoprotein | 40.67 (8.48) | 40.02 (9.34) | 0.71 | 40.88 (10.58) | 39.64 (8.06) | 0.61 |
|  | Low Density Lipoprotein | 106.58 (38.88) | 109.20 (35.24) | 0.60 | 103.36 (39.85) | 112.39 (32.51) | 0.34 |
|  | Gamma Glutamil Aminotransferase | 19.42 (11.56) | 17.14 (11.60) | 0.55 | 18.56 (12.08) | 16.92 (11.26) | 0.59 |

Boldface indicates statistical significance (*p*<0.05). Abbreviations: BDI: Beck depression inventory; BMI: body mass index, calculated as weight in kilograms divided by height in meters squared; DBP: diastolic blood pressure; HRQoL: health-related quality of life; SBP: systolic blood pressure; SF-36: 36-item short form health survey.

^a^ *P* value. Groups were compared using a paired t-test. A positive value in the following variables represents a better health state: subscales of the HRQoL questionnaire (SF-36) and in high-density lipoprotein. In all other cases, a negative value represents a better health state.

**Table S2.** Mixed-effects regression results for weight change

|  | | **Intention-to-treat analysis (n=62)^a^** | | | | **Completers analysis (n=37)^b^** | | | |
| --- | --- | --- | --- | --- | --- | --- | --- | --- | --- |
| **Variable** | | **Coefficient** | **Standard error** | **95% confidence interval** | ***P*** | **Coefficient** | **Standard error** | **95% confidence interval** | ***P*** |
| **Treatment (1=DPP-web)** | | 0.78 | 0.76 | (-0.72, 2.28) | 0.31 | 1.25 | 1.09 | (-0.89, 3.39) | 0.25 |
| **Time** | |  |  |  |  |  |  |  |  |
|  | 3 months | 0.57 | 0.64 | (-0.68, 1.82) | 0.37 | 0.94 | 1.24 | (-0.71, 2.59) | 0.27 |
|  | 6 months | 0.78 | 0.64 | (-0.47, 2.03) | 0.22 | 0.84 | 1.24 | (-0.81, 2.9) | 0.32 |
| **Interaction** | |  |  |  |  |  |  |  |  |
|  | DPP-web x 3 months | -3.65 | 0.90 | (-5.42, -1.89) | <0.01 | -5.39 | -4.34 | (-7.83, -2.96) | <0.01 |
|  | DPP-web x 6 months | -5.50 | 0.90 | (-7.26, -3.73) | <0.01 | -6.78 | -5.46 | (-9.21, -4.34) | <0.01 |
| **Pain perception at baseline (score)** | | -0.04 | 0.01 | (-0.07, -0.02) | <0.01 | -0.06 | 0.02 | (-0.09, -0.03) | <0.01 |
| **Health transition at baseline (score)** | | 0.00 | 0.01 | (-0.02, 0.03) | 0.80 | 0.00 | 0.02 | (-0.04, 0.03) | 0.89 |
| **Intercept** | | 2.44 | 0.87 | (0.73, 4.15) | <0.01 | 3.70 | 1.38 | (-0.09, -0.03) | <0.01 |
| **Log likelihood** | | -455.26 |  |  | 0.01 | -278.79 |  |  | 0.01 |
| **Standard deviation (Intercept)** | | 1.39 | 0.28 | (0.94, 2.05) |  | 1.52 | 0.38 |  |  |
| **Standard deviation (Residual)** | | 2.51 | 0.16 | (2.22, 2.84) |  | 2.66 |  |  |  |
| **chibar^2^(01)** | | 9.27 |  |  | <0.01 | 6.12 |  |  | <0.01 |

This table presents the findings of a mixed-effects regression analysis aimed at evaluating the impact of the DPP-web treatment on weight changes over a 6-month period considering both the results obtained with the intention-to-treat and the completers analyses.

^a^ The intention-to-treat analysis followed the intention-to-treat principle using Markov Chain Monte Carlo method for multiple imputations to estimate missing values (14 missing values at three months and 25 at six months). It shows significant interaction effects, specifically "DPP-web x 3 months" and "DPP-web x 6 months," both indicating significant reductions in weight change compared to the wait-list control (*P* < 0.01). Additionally, the baseline pain perception variable displays a negative association: for every unit increase in the baseline pain perception score, indicating lower perceived pain, there is a corresponding improvement in weight loss of -0.04 kg (*P* < 0.01). This is in contrast to the non-significant impact of health transition at baseline (*P* = 0.80). The likelihood ratio test (chibar2(01)) confirms the superiority of the mixed-effects model over a simpler linear model (*P* < 0.01). Overall, these results emphasize the significant impact of the DPP-web treatment, particularly when interacting with time, while highlighting the substantial role of baseline pain perception in weight changes.

^b^ The completers analysis considered all participants who attended measurements at six months (17 for the DPP-web group and 20 for the control group). This evaluation echoed the findings from the intention to treat analysis, revealing significant interaction effects within the "DPP-web x 3 months" and "DPP-web x 6 months" variables, both denoting significant reductions in weight change (*P* < 0.01). Additionally, we identified a consistent negative association with the baseline pain perception variable: for every unit increase in the baseline pain perception score, suggesting reduced perceived pain, there was a corresponding decrease of -0.06 kg (*P* < 0.01). In contrast, the influence of health transition at baseline remained non-significant (*P* = 0.89). The likelihood ratio test (chibar2(01)) reconfirmed the superiority of the mixed-effects model over a simpler linear model (*P* < 0.01).

**Table S3.** Changes in body weight and other variables at 3 and 6 months of intervention by completers analysis^a^ (n=48 at 3 months and 37 at 6 months)

| **Outcome** | | **DPP-web at 3 months** ^b^  **(n=25)** | **Control**  **at 3 months** ^b^  **(n=23)** | **Treatment effect at 3 months** ^c^ | **DPP-web at 6 months** ^b^ **(n=17)** | **Control at 6 months** ^b^ **(n=20)** | **Treatment effect at 6 months** ^c^ |
| --- | --- | --- | --- | --- | --- | --- | --- |
|  | | **Mean (SD)** | **Mean (SD)** | **Mean (95%CI); P value** | **Mean (SD)** | **Mean (SD)** | **Mean (95%CI); P value** |
| **Body weight (kg)** | | -3.75 (3.79)* | 0.97 (2.79) | -4.73 (-6.65, -2.80); **≤0.01** | -5.94 (6.40)* | 0.84 (2.98) | -6.78 (-10.28, -3.28); **≤0.01** |
| **Body weight (%)** | | -3.94 (3.65)* | 1.22 (2.79) | -5.16 (-7.06, -3.27); **≤0.01** | -5.87 (5.85)* | 0.90 (3.47) | -6.77 (-10.10, -3.45); **≤0.01** |
| **Body fat (%)** | | -1.66 (1.87)* | 0.06 (1.43) | -1.72 (-2.69, -0.75); **≤0.01** | -2.81 (2.42)* | -0.05 (1.49) | -2.76 (-4.08, -1.44); **≤0.01** |
| **Body mass index (kg/m^2^)b** | | -1.32 (1.26)* | 0.40 (0.94) | -1.72 (-2.38, -1.07); **≤0.01** | -2.05 (2.11)* | 0.30 (1.07) | -2.35 (-1.44, -0.11); **≤0.01** |
| **Waist circumference (cm)** | | -4.60 (4.20)* | -0.15 (3.26) | -4.45 (-6.65, -2.25); **≤0.01** | -7.00 (5.79)* | -0.70 (3.79) | -6.30 (-9.52, -3.08); **≤0.01** |
| **Systolic blood pressure (mm/Hg)** | | -0.96 (9.33) | 2.65 (10.52) | -3.61 (-9.38, 2.15); 0.21 | -5.24 (10.62) | 0.15 (10.69) | -5.39 (-12.52, 1.75); 0.13 |
| **Diastolic blood pressure (mm/Hg)** | | -3.60 (6.74)* | 0.91 (6.40) | -4.51 (-8.34, -0.69); **0.02** | -3.24 (7.63) | 0.30 (10.28) | -3.54 (-9.67, 2.60); 0.25 |
| **Depressive symptoms: BDI (score)** | | -5.96 (5.86)* | -0.09 (6.17) | -5.87 (-9.37, -2.38); **≤0.01** | -4.35 (7.27)* | -0.15 (9.82) | -4.20 (-10.06, 1.66); 0.15 |
| **HRQoL:SF-36 (score)** | |  |  |  |  |  |  |
|  | Physical functioning | 8.20 (10.19)* | -0.65 (12.28) | 8.85 (2.32, 15.39); **≤0.01** | 7.35 (18.80) | 1.00 (14.47) | 6.35 (-4.76, 17.46); 0.25 |
|  | Role limitations due to physical problems | 12.00 (24.07)* | 1.14 (41.89) | 10.86 (-8.91, 30.64); 0.27 | 10.29 (23.48) | 7.89 (46.44) | 2.40 (-23.00, 27.80); 0.85 |
|  | Bodily pain | 5.30 (22.22) | 5.43 (15.57) | -0.13 (-11.38, 11.11); 0.98 | 5.44 (14.85) | 7.50 (22.55) | -2.06 (-15.06, 10.94); 0.75 |
|  | Social functioning | 0.39 (28.66) | 4.24 (23.81) | 2.76 (-12.62, 18.14); 0.72 | 9.56 (26.71) | 1.88 (27.59) | 7.68 (-10.53, 25.89); 0.40 |
|  | Mental health | 4.64 (19.17) | 1.22 (14.14) | 3.42 (-6.44, 13.28); 0.49 | 3.06 (18.58) | 5.45 (16.00) | -2.39 (-13.93, 9.14); 0.68 |
|  | Role limitations due to emotional problems | 10.68 (28.43) | -2.89 (34.69) | 13.57 (-4.80, 31.93); 0.14 | 15.69 (51.53) | 13.36 (33.15) | 2.34 (-26.16, 30.84); 0.87 |
|  | Vitality | 11.20 (17.69)* | 4.35 (17.14) | 6.85 (-3.29, 16.99); 0.18 | 11.18 (18.16)* | 4.75 (16.34) | 6.43 (-5.09, 17.94); 0.27 |
|  | General health perceptions | 13.60 (18.29)* | 3.26 (14.89) | 10.34 (0.60, 20.08); **0.04** | 17.35 (14.80)* | 4.50 (16.29) | 12.85 (2.39, 23.32); **0.02** |
|  | Health transition | 23.00 (26.93)* | 8.70 (23.37) | 14.30 (-0.40, 29.01); 0.06 | 25.00 (29.32)* | 7.50 (18.32) | 17.50 (1.44, 33.56); 0.03 |
| **Biochemical variables** | |  |  |  |  |  |  |
|  | Fasting glucose (mg/dL) | -1.08 (13.58) | -3.77 (9.80) | 2.69 (-4.35, 9.74); 0.45 |  |  |  |
|  | Total cholesterol (mg/dL) | 5.00 (28.35) | 1.86 (29.70) | 3.14 (-13.93, 20.20); 0.71 |  |  |  |
|  | Tryglicerides (mg/dL) | -16.92 (45.93) | -25.55 (48.08)* | 8.63 (-19.01, 36.26); 0.53 |  |  |  |
|  | High density lipoprotein (mg/dL) | -0.08 (7.37) | 0.00 (8.06) | -0.08 (-4.61, 4.45); 0.97 |  |  |  |
|  | Low density lipoprotein (mg/dL) | -1.16 (33.12) | -3.05 (32.65) | 1.89 (-17.49, 21.26); 0.85 |  |  |  |
|  | Gamma glutamyltransferase (mg/dL) | 0.16 (10.90) | 2.91 (5.22) | -2.75 (-7.71, 2.21); 0.27 |  |  |  |

Boldface indicates statistical significance (*p*<0.05). Abbreviations: BDI: Beck depression inventory; BMI: body mass index, calculated as weight in kilograms divided by height in meters squared; DBP: diastolic blood pressure; HRQoL: health-related quality of life; SBP: systolic blood pressure; SF-36: 36-item short form health survey.

^a^ All analyses were conducted considering the participants who attended the measurements at 3 and 6 months, respectively.

^b^ The change at 3 and 6 months was calculated as the final value minus the baseline value.

^c^ Treatment effect is defined as the change in the DPP-web group minus the change in the wait-waiting list control group. Data are presented as means (95% confidence intervals); *P* value. Groups were compared using a paired t-test. A positive value in the following variables represents an improvement: subscales of the HRQoL questionnaire (SF-36) and in high-density lipoprotein. In all other cases, a negative value represents an improvement

*Asterisk indicates statistical significance (*P* ≤ 0.05) within the group assessing differences using independent samples t-test

**Table S4.** Success in achieving the weight loss goals at 3 and 6 months according to treatment group

| **Analysis** | **Weight Loss** | **Number of participants that achieve weight loss goal at 3 months** | | ***P*** ^a^ | **Number of participants that achieve weight loss goal at 6 months** | | ***P*** ^a^ |
| --- | --- | --- | --- | --- | --- | --- | --- |
|  |  | **Intervention**  n (%) | **Control**  n (%) |  | **Intervention**  n (%) | **Control**  n (%) |  |
| **Intention to treat** ^b^ | >5% | 8 (26) | 0 (0) | **0.01** | 11 (35) | 0 (0) | **<0.01** |
|  | >10% | 2 (6) | 0 (0) | 0.15 | 5 (16) | 0 (0) | **0.02** |
| **Completers** ^c^ | >5% | 8 (32) | 0 (0) | **<0.01** | 7 (41) | 0 (0) | **<0.01** |
|  | >10% | 2 (8) | 0 (0) | 0.17 | 4 (24) | 0 (0) | **0.02** |

^a^ All analyses were conducted considering the participants who attended the measurements at 3 and 6 months, respectively.

^b^ Chi-square test.

^c^ The analysis followed the intention-to-treat principle using Markov Chain Monte Carlo method for multiple imputations to estimate missing values (14 missing values at three months and 25 at six months).

^d^ The completers analysis considered all participants who attended measurements at six months (17 for the DPP-web group and 20 for the control group).
